# miR-101-3p represses the migratory and invasive abilities of ovarian cancer cells

**DOI:** 10.1186/s12885-025-15280-9

**Published:** 2025-12-11

**Authors:** Li Chen, Haizhou Ji, Zuolian Xie, Yiting Lin, Ling Li, Liang Lin, An Lin

**Affiliations:** Department of Gynecology, Clinical Oncology School of Fujian Medical University, Fujian Cancer Hospital, No. 420, Fuma Road, Fuzhou, 350014 China

**Keywords:** MiR-101-3p, Ovarian cancer, Invasion, Migration, C-X-C motif ligand 8

## Abstract

**Background:**

The study aimed to assess the effects of miR-101-3p on the migratory and invasive abilities of ovarian cancer cells, specifically in the SKOV3 and OVCAR-3 cell lines.

**Methods:**

The expression of miR-101-3p was overexpressed in SKOV3 and OVCAR-3 ovarian cancer cell lines via mimic transfection. The effects on cell viability, migration, and invasion were evaluated using CCK8, wound healing, and Transwell assays, respectively. RNA sequencing was subsequently performed to identify differentially expressed genes.

**Results:**

Overexpression of miR-101-3p significantly decreased cell viability, migration, and invasion in both SKOV3 and OVCAR-3 cells. Transcriptomic analysis identified numerous differentially expressed genes, including FN-1 and CXCL8. Intriguingly, while miR-101-3p suppressed FN-1 mRNA in SKOV3 cells, it led to an increase in both FN-1 and CXCL8 mRNA levels in OVCAR-3 cells, despite inhibiting their invasive phenotype.

**Conclusions:**

Our study confirms that miR-101-3p acts as a tumor suppressor in ovarian cancer. The paradoxical regulation of downstream gene transcripts highlights a complex, cell-type-specific regulatory network. This suggests that the overall phenotypic effect of miR-101-3p is an integrated outcome of modulating multiple targets, rather than a linear pathway, and underscores its potential as a therapeutic target requiring further mechanistic study.

**Clinical trial number:**

Not applicable.

**Supplementary Information:**

The online version contains supplementary material available at 10.1186/s12885-025-15280-9.

## Background

Ovarian cancer represents the deadliest gynecologic cancer, with a lifetime risk approximating 1 in 50 to 70 women [1, 2]. According to GLOBOCAN 2020, there were about 313,959 newly diagnosed ovarian cancer cases in 2020, with 207,252 related deaths [3]. Individuals aged 60–64 years are the most affected, with majority of ovarian cancer diagnoses occurring in individuals over 50 years of age [1, 2]. Ovarian cancer diagnosis is difficult, as there are no reliable screening tests and symptoms for this malignancy, which results in most diagnoses occurring at late disease stages [4, 5]. Unfortunately, even with the best care, 5-year overall survival in ovarian cancer is low, at around 45% for all cases, and 89%, 70%, 36%, and 17% in individuals with stage I to IV disease, respectively [6]. The risk of ovarian cancer recurrence is high despite the best treatments, and increases with disease stage (10%, 30%, 70–90% and 90–95% in stage I-IV disease, respectively) [2]. Hence, the molecular mechanisms of ovarian cancer should be further examined, which may help improve treatments and prevent recurrence.

Tumor metastasis and recurrence are tightly associated with the epithelial-mesenchymal transition (EMT) [7–9]. After EMT, tumor cells become drug-resistant and acquire a higher proliferative potential as well as migratory and invasive abilities [7–9]. Drug resistance and increased proliferation promote cancer recurrence [7–9]. In addition, during EMT, epithelial cell genes (e.g., E-cadherin) are downregulated while mesenchymal cell genes (e.g., Snail, Slug, and Vimentin) are upregulated [10, 11]. Tumor cell migration and invasion are central features contributing to the spread of tumor cells to adjacent tissues and structures, eventually leading to metastatic spread [12]. EMT reduces cell cohesion [10, 11], and tumor cells gain the ability to detach from the primary tumor, spread via the extracellular matrix, and enter into the peripheral or lymphatic circulation, from which they reach distant sites [13]. Consequently, preventing migration and invasion is a promising approach to managing tumor progression and metastatic spread [14, 15].

Many miRNAs are involved in ovarian cancer [16] and cancer in general [17]. Among the large number of known candidates, miRNA-101-3p is promising as it suppresses proliferation, invasion, and chemoresistance in multiple cancer types, e.g., salivary gland [18], lung [19, 20], kidney [21], cervix [22], and retinoblastoma [23]. Still, the exact mechanism of miR-101-3P in ovarian cancer is mostly undefined, especially regarding its role in disease recurrence and metastasis.

Therefore, the present work aimed to examine miR-101-3p’s effects on the migratory and invasive capabilities of ovarian cancer SKOV3 (non-serous carcinomas; high migratory behavior) and OVCAR-3 (high-grade serous carcinomas; spreading broadly in the peritoneal cavity) cells [24]. Cultured SKOV3 and OVCAR-3 cells were examined by qRT-PCR for miR-101-3p amounts. After miR-101-3p overexpression, cell viability was assessed by the CCK8 assay. Then, migration and invasion were quantitated by the wound healing and Transwell assays, respectively. The cells next underwent mRNA high-throughput sequencing. The findings should provide valuable insights into miR-101-3p’s impacts on ovarian cancer recurrence and metastasis. Such knowledge could eventually translate into new treatments.

## Methods

### Reagents and instruments

SKOV3 and OVCAR3 cells were provided by Procell Life Science & Technology (China). RPMI-1640 complete medium, RPMI-1640 incomplete medium, complete McCoy’s 5 A medium, and incomplete McCoy’s 5 A medium were from KeyGen Biotech (China). Trypsin-EDTA digestion solution and crystal violet staining solution were from Solarbio Science & Technology (China). The TRIzol reagent, miRNA extraction kit, and ultra-pure RNA extraction kit were from cwBiotech (China).

The experimental instruments included a CO_2_ incubator (BPN-80CW, Shanghai Yiheng Scientific Instruments, China), fully-automated microplate reader (WD-2012B, Liuyi, China), CX41 microscope (Olympus, Japan), standard PCR thermal cycler (TC-EA, Hangzhou Borui Technology, China), and fluorescence PCR machine (CFX Connect™ real-time, Berle Life Science Medical Products (Shanghai), China).

### Cell culture and treatment

SKOV3 and OVCAR3 cells underwent culture at 37 °C in RPMI-1640 complete medium (plus 10% FBS and 1% penicillin/streptomycin) with 5% CO_2_. To investigate miR-101-3p’s effect on ovarian cancer cells, Lipferon 3000 was used to transfect miR-101-3p mimic (ZvastBio) and its negative control (NC) into the cells, constituting the miR-101-3p mimic and mimic NC groups, respectively. Subsequent tests were conducted after successful transfection.

### Ovarian cancer tissue samples

Ovarian cancer and adjacent noncancerous tissues (*N* = 30) were obtained from ovarian cancer cases undergoing elective resection. The sampling had approval from the Ethics Committee of Fujian Cancer Hospital (#SQ2021-001-01). All patients provided signed informed consent for sampling.

### qRT-PCR

Total RNA extraction from ovarian cancer cells utilized the TRIzol reagent. The miRNAs were extracted with the RNA Ultra-pure Extraction Kit/miRNA Ultra-pure Extraction Kit. Then, mRNA amounts and purity were assessed with a Nanodrop (OD_260_/OD_280_). cDNA synthesis was performed with the RNA reverse transcription kit/miRNA reverse transcription kit, followed by fluorescence quantitative PCR employing the fluorescence PCR machine. The reaction steps were (1) pre-denaturation (95 °C, 10 min), (2) denaturation (95 °C, 10 s), and (3) 40 cycles of annealing (58 °C, 30 s) and extension (72 °C, 30 s). The 2^−ΔΔCt^ method was utilized for the analysis of data, normalized to β-actin/U6 expression. Table 1 shows the primer sequences used in this study, including HMCN1, which was selected based on prior literature indicating its potential role in ovarian cancer [25].

**Table 1:** Primers used for qRT-PCR

Primer names	Forward (5’->3’)	Reverse (5’->3’)
miR-101-3p	GCG CGC GTA CAG TAC TGT GAT A	AGT GCA GGG TCC GAG GTA TT
U6	CTC GCT TCG GCA GCA CA	AAC GCT TCA CGA ATT TGC GT
β-actin	TGG CAC CCA GCA CAA TGA A	CTA AGT CAT AGT CCG CCT AGA AGC A
FN-1	GAC GAC TCC CTT TTC TCC TCT TG	TGG CTC ATC TCC CTC CTC ACT
CXCL8	GAC ATA CTC CAA ACC TTT CCA C	ACT TCT CCA CAA CCC TCT GC
HMCN1	CTG CCC ACC TGG CTA TCA AC	CAG TTC TTG AGG CAG AAC CCT

### CCK8 assay

Cells from the two transfection groups were collected at 0, 24, 48, and 72 h for analysis, respectively. The cells were transferred in the same culture medium (100 µL/well) to a 96-well plate. After transfection, the CCK8 reagent (10 µL/well) was added, followed by a 2-h incubation and OD reading at 450 nm using a plate reader.

### Wound healing assay

At a cell confluence of more than 90% in each group, a line was made on each well with a 200-µL pipette tip. Then, three PBS washes were performed, and serum-free medium was supplemented into all wells. The generated wound was photographed. The cells were next placed into the incubator for 24 and 48 h, respectively, and photographed. Wound areas were assessed at 24 and 48 h, respectively, relative to the 0 h control, to quantitate cell migration. The degree of wound healing was evaluated by calculating the healing rate as the area of cell migration after a period of time by the area before cell migration.

### Transwell invasion assay

The invasive ability of cells was assessed using Transwell chambers (8 μm pore size) pre-coated with Matrigel. After transfection, 5 × 10⁴ cells were seeded into the upper chamber in serum-free medium. The lower chamber was filled with complete medium containing 10% FBS. After 24 h of incubation at 37 °C, cells that had invaded to the lower surface of the membrane were fixed, stained with 0.1% crystal violet, and counted. For quantification, the stain was dissolved in 33% acetic acid, and the absorbance was measured at 562 nm.

### mRNA high-throughput sequencing

Total RNA extraction from cells was carried out employing the TRIzol reagent. This was followed by mRNA purification using the RNA purification kit. mRNA amounts and purity were determined with a UV-visible spectrophotometer (OD260/OD280), and RNA integrity was evaluated by agarose gel electrophoresis. The transcriptome was sequenced on an Illumina Hiseq™ platform. Differential expression analysis was performed using the DESeq2 R package. Genes meeting the criteria of an adjusted P-value < 0.05 and |log2(FoldChange)| ≥ 1 were identified as differentially expressed genes (DEGs), which are presented in the volcano plot (Fig. 4). To further explore the biological implications, KEGG and GO enrichment analyses were performed, highlighting pathways that may be regulated by miR-101-3p. Raw sequencing data have been deposited in the NCBI Sequence Read Archive under accession number PRJNA1174342.

### Statistical analysis

SPSS 20.0 (SPSS, USA) was employed for statistics. All assays were independently repeated three times. The assays were performed thrice. Quantitative data are mean ± standard deviation. The independent samples t-test was carried out to determine between-group differences in quantitative data. For multi-group comparisons, one-way ANOVA followed by Tukey’s post hoc test was applied. Two-sided *P* < 0.05 reflected statistical significance.

## Results

### miR-101-3p is downregulated in ovarian cancer tumor tissues

**Fig. 1:**
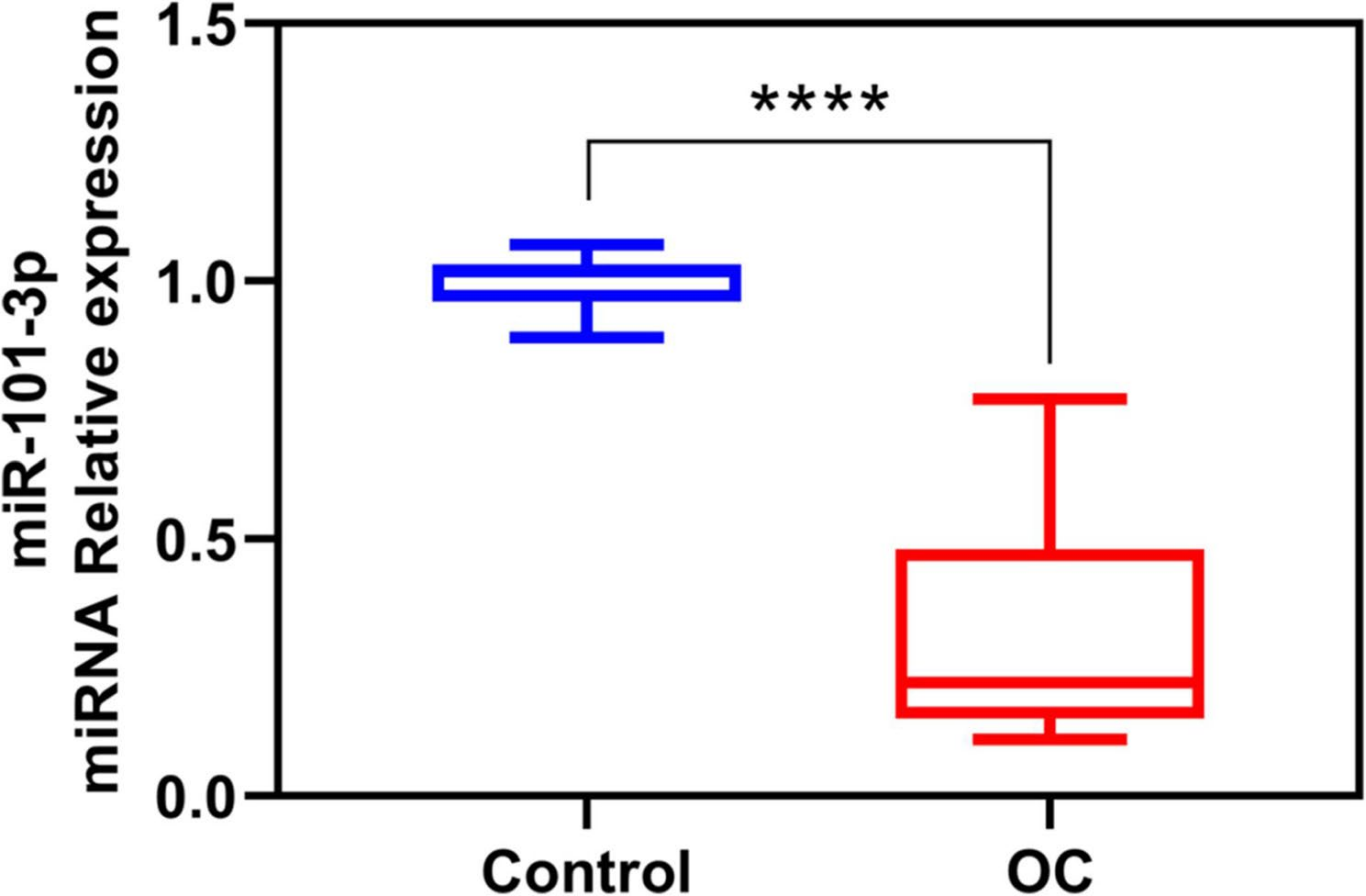
qPCR analysis of miR-101-3p in the OC (ovarian cancer tumor tissues) and Control (adjacent non-cancerous tissues) groups. ****P < 0.0001 versus the control group. *N* = 30. All experiments were independently repeated three times

In our clinical cohort of 30 paired ovarian cancer and adjacent non-cancerous tissues, miR-101-3p expression was markedly reduced in tumor tissues (*P* < 0.0001, Fig. 1).

### miR-101-3p reduces viability in the SKOV3 and OVCAR3 cell lines

**Fig. 2:**
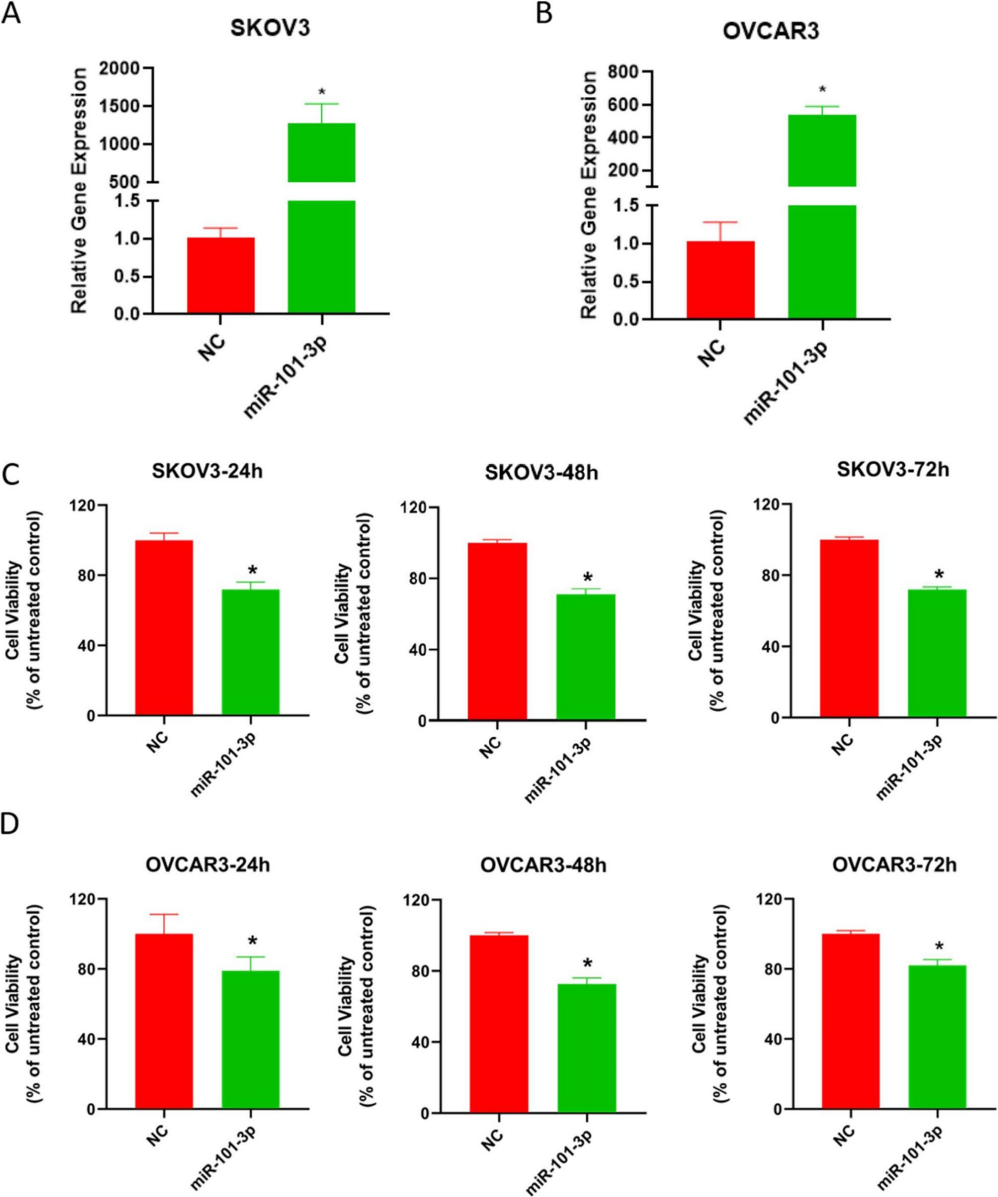
Effects of miR-101-3p on the viability of ovarian cancer SKOV3 and OVCAR3 cells. (**A**-**B**) qPCR verification of miR-101-3p mimic transfection in SKOV3 and OVCAR3 cells. *P < 0.05 versus the NC group. (**C**-**D**) Effects of miR-101-3p on the viability of ovarian cancer SKOV3 and OVCAR3 cells at 24 h, 48 h and 72 h. *P < 0.05 versus the NC group. All experiments were independently repeated three times

miR-101-3p was starkly upregulated in both SKOV3 and OVCAR3 cell lines upon transfection with miR-101-3p mimic in comparison with the NC group (*P* < 0.05), confirming the successful overexpression (Fig. 2A-B). Meanwhile, significantly reduced viability was detected in SKOV3 and OVCAR3 cell lines after transfection with miR-101-3p mimic at 24, 48, and 72 h versus the NC group (*P* < 0.05, Fig. 2C-D).

### miR-101-3p decreases the migratory abilities of SKOV3 and OVCAR3 cells

**Fig. 3:**
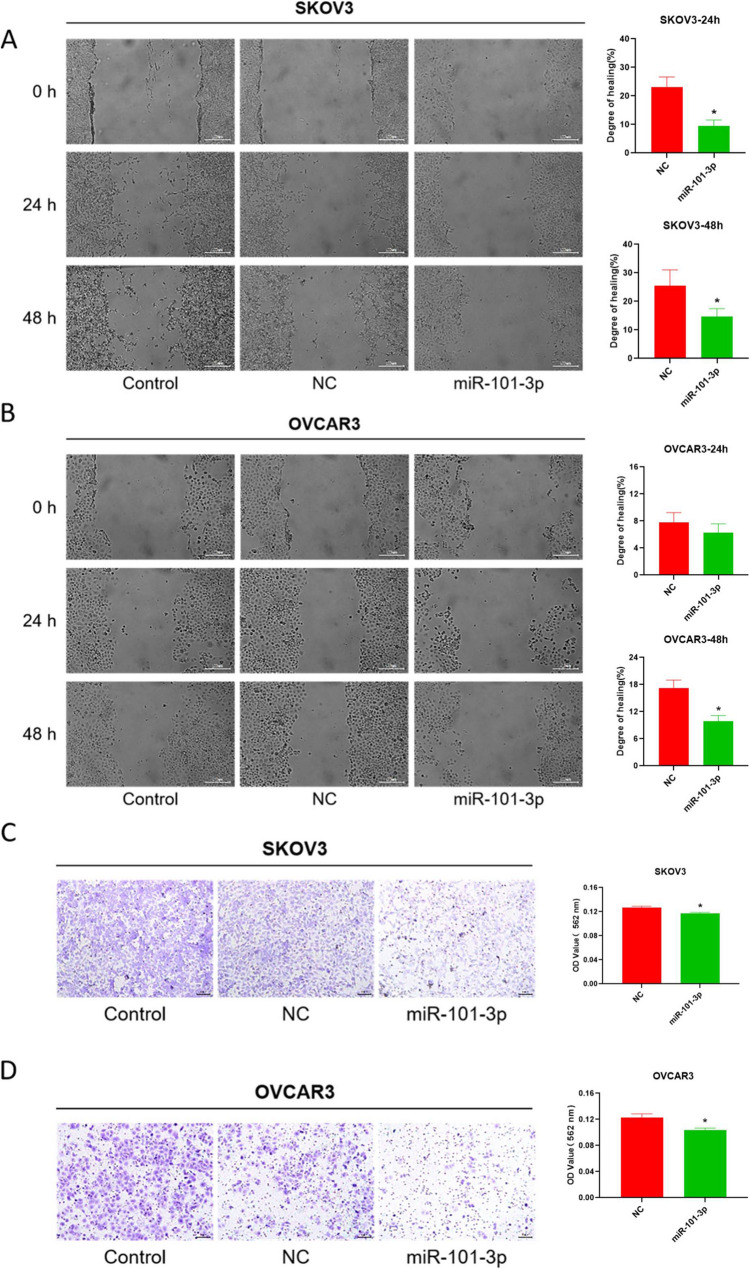
Effects of miR-101-3p on the migration and invasion of ovarian cancer SKOV3 and OVCAR3 cells. (**A**-**B**) Effects of miR-101-3p on the migratory abilities of ovarian cancer SKOV3 and OVCAR3 cell lines at 24 h and 48 h. *P < 0.05 versus the NC group. (**C**-**D**) Effects of miR-101-3p on the invasive abilities of ovarian cancer SKOV3 and OVCAR3 cell lines. *P < 0.05 versus the NC group. All experiments were independently repeated three times

The migratory capability of ovarian cancer SKOV3 cells was significantly reduced at 24 and 48 h following transfection with miR-101-3p mimic in comparison with NC-transfected cells (*P* < 0.05, Fig. 3A-B). Similarly, the migratory ability of OVCAR3 cells was markedly reduced at 48 h following transfection with miR-101-3p mimic (*P* < 0.05). The invasive abilities of both SKOV3 and OVCAR3 cells were remarkably reduced at 48 h following transfection with miR-101-3p mimic in comparison with NC-transfected cells (Fig. 3C-D, *P* < 0.05).

### miR-101-3p induces significant changes in the transcriptomes of SKOV3 and OVCAR3 cells

**Fig. 4:**
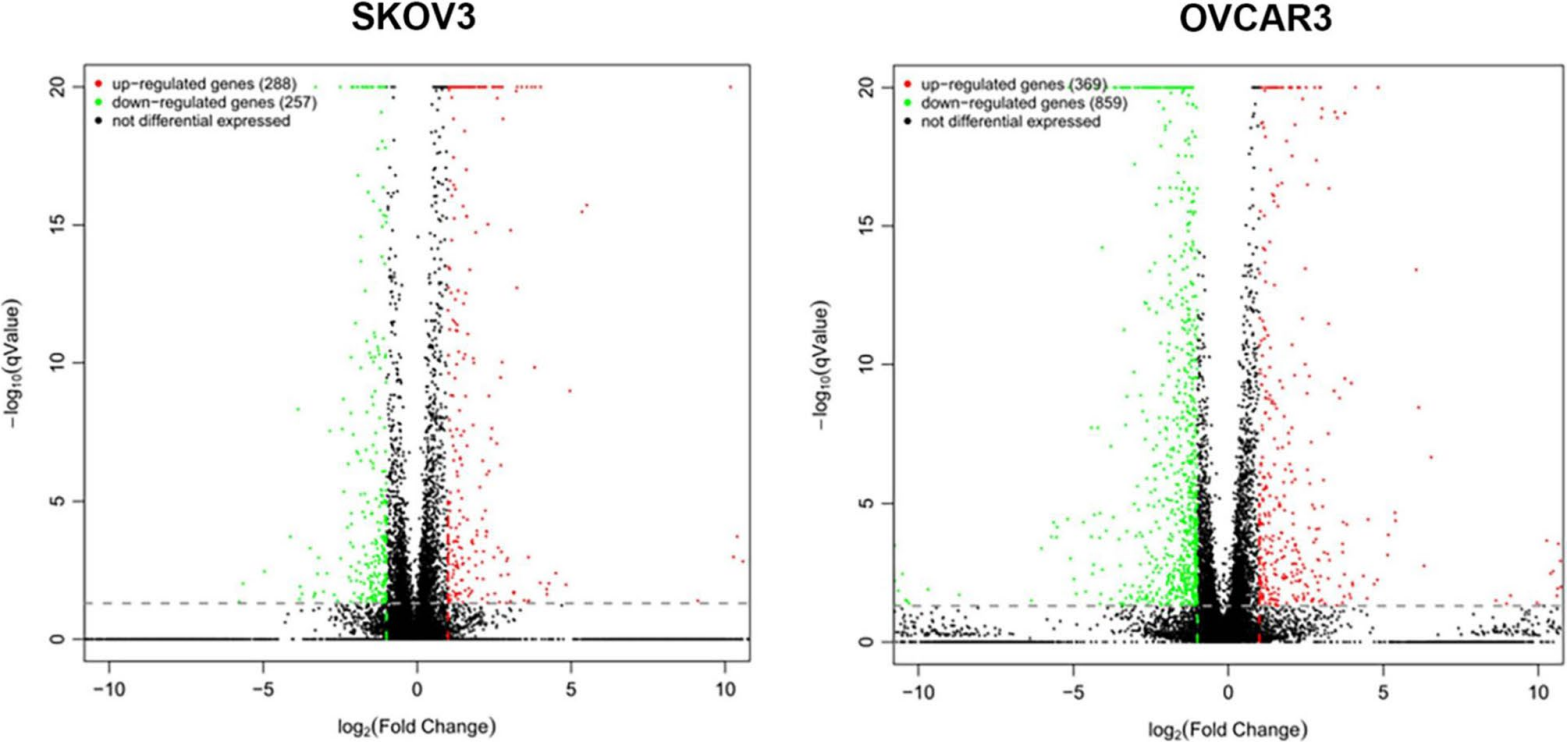
Volcano plots of differentially expressed genes in SKOV3 and OVCAR3 cells after miR-101-3p mimic transfection. Green and red dots represent upregulated and downregulated genes, respectively

**Fig. 5:**
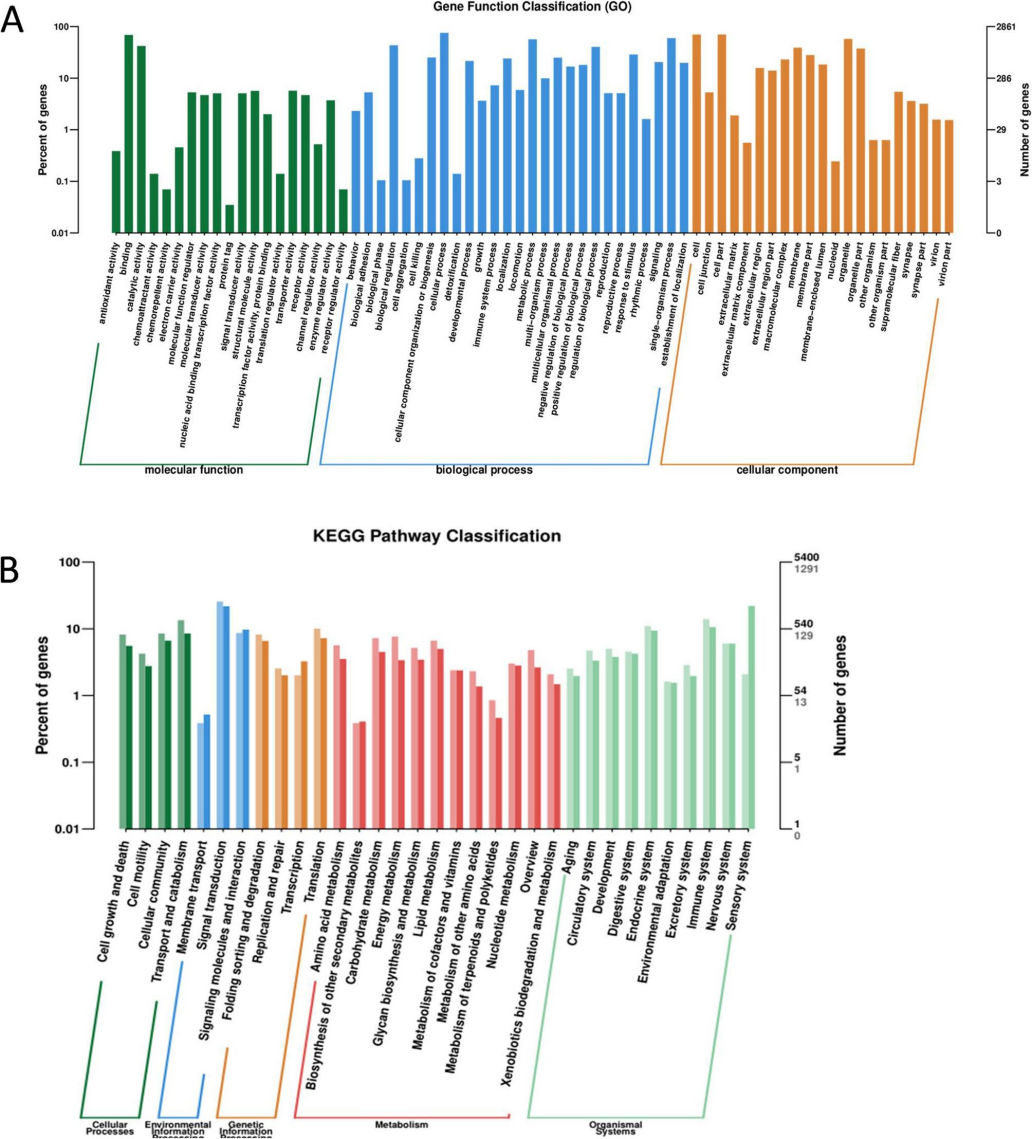
Gene ontology (GO) and Kyoto Encyclopedia of Genes and Genomes (KEGG) analyses of genes affected by miR-101-3p overexpression. (**A**) GO annotation of differentially expressed mRNAs. (**B**) KEGG pathway classification of differentially expressed mRNAs

Figure 4 shows volcano plots depicting DEGs in the SKOV3 and OVCAR3 cell lines. A Venn diagram of DEGs between the two cell lines was added (Supplementary Figure S1), showing their overlap, followed by KEGG and GO analyses of the common DEGs (Supplementary Figure S2). To summarize systems-level relationships among DEGs, a protein–protein interaction (PPI) network was constructed (Supplementary Figure S3). The genes obtained from the sequencing data were subjected to GO classification analysis using the ClusterProfiler R package (Fig. 5), to analyze their involvement in the biological process, cellular component, and molecular function categories. The horizontal axis represents the second-level classification of GO terms, while the vertical axes display gene numbers (right) and the percentages of DEGs among annotated genes (left) in various categories. Different colors represent distinct orthologs. As depicted in Fig. 5A, the mRNAs most altered by miR-101-3p belonged to binding and catalytic activity in biological processes; cellular process, metabolic process, and single-organism process in cellular components; and cell, cell part, and organelle in molecular functions.

After GO annotation, the genes were classified considering their associations with KEGG metabolic pathways. The horizontal axis shows the names of the associated pathways, while the vertical axis displays the number of genes annotated in each pathway (Fig. 5B). In cellular processes, miR-101-3p was implied in cell growth and death, cell motility, cellular community, and transport and catabolism. In environmental information processing, miR-101-3p contributed to signal transduction, membrane transport, and signaling molecules and interactions. In genetic information processing, miR-101-3p was involved in folding sorting and degradation, replication and repair, translation, and transcription. In metabolism, miR-101-3p was implied in amino acid metabolism, energy metabolism, glycan biosynthesis and metabolism, lipid metabolism, metabolism of cofactors and vitamins, metabolism of terpenoids and polyketides, nucleotide metabolism, overview, xenobiotics biodegradation and metabolism, and biosynthesis of other secondary metabolites. Finally, in organismal systems, miR-101-3p was implied in aging, circulatory system, development, digestive system, endocrine system, environmental adaptation, excretory system, immune system, nervous system, and sensory system.

### miR-101-3p downregulates and upregulates the FN-1 and CXCL8 genes in SKOV3 cells but increases the amounts of both mRNAs in OVCAR3 cells

**Fig. 6:**
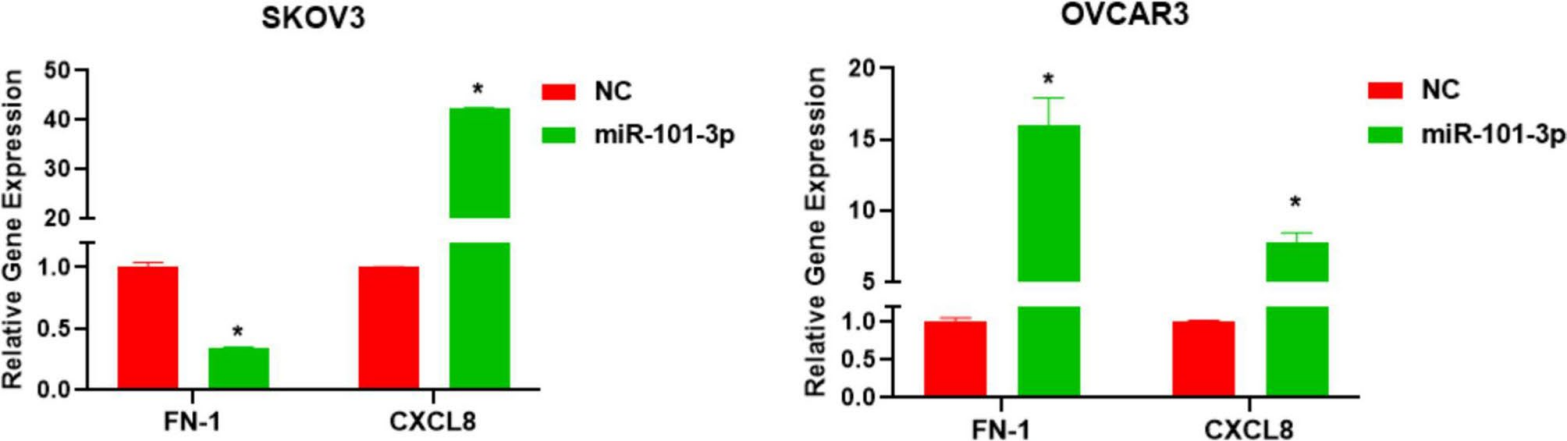
Validation of FN-1 and CXCL8 mRNA expression in ovarian cancer cell SKOV3 and OVCAR3 lines by qPCR. *P < 0.05 versus the NC group. All experiments were independently repeated three times

Based on the results from high-throughput sequencing, FN-1 and CXCL8 were identified as DEGs upon miR-101-3p mimic transfection. In SKOV3 cells, versus NC-transfected cells, FN-1 mRNA amounts were remarkably lowered in miR-101-3p-transfected cells (*P* < 0.05), while CXCL8 mRNA expression was significantly elevated (*P* < 0.05, Fig. 6). In OVCAR3 cells, miR-101-3p transfection starkly increased FN-1 and CXCL8 mRNA amounts in comparison with NC-transfected cells (*P* < 0.05), corroborating high-throughput sequencing data. A comprehensive list of DEGs is provided in Supplementary Table S1. Predicted target analyses using TargetScan, miRDB, and TarBase databases indicated that both FN-1 and CXCL8 are potential direct targets of hsa-miR-101-3p (Supplementary Table S2). Survival analysis using the Kaplan–Meier Plotter revealed that high FN-1 expression was associated with poorer overall survival, whereas elevated CXCL8 expression correlated with a more favorable prognosis (Supplementary Figure S4).

## Discussion

Ovarian cancer remains a significant cause of mortality among gynecologic malignancies, largely due to late-stage diagnosis and high rates of metastasis and recurrence. MicroRNAs are crucial regulators in oncogenesis, and miR-101-3p has been identified as a tumor suppressor in various cancers. Our study corroborates these findings, demonstrating that miR-101-3p expression is significantly lower in ovarian cancer tissues compared to adjacent non-cancerous tissues [18–23]. Furthermore, restoring miR-101-3p expression in ovarian cancer cell lines SKOV3 and OVCAR-3 significantly decreased cell viability, migration, and invasion. These results align with the established role of miR-101-3p in mitigating the malignant phenotype of cancer cells. The epithelial-mesenchymal transition (EMT) is pivotal in conferring migratory and invasive capabilities to tumor cells. Previous studies have linked miR-101-3p to EMT inhibition, for instance, by targeting ZEB1 [26] and affecting FN1 expression in serous ovarian cancer [27]. Our findings of reduced migration and invasion upon miR-101-3p overexpression are consistent with an anti-EMT effect. The Gene Ontology (GO) and Kyoto Encyclopedia of Genes and Genomes (KEGG) analyses from our mRNA sequencing further indicated that miR-101-3p broadly influences cellular processes integral to cell motility, growth, and death.

A significant and perplexing observation in our study is the upregulation of FN-1 and CXCL8 mRNA levels in OVCAR-3 cells following miR-101-3p mimic transfection, despite a concurrent reduction in migratory and invasive capacities. This paradoxical finding, where pro-tumorigenic gene transcripts are elevated while the malignant phenotype is suppressed, suggests that the functional impact of miR-101-3p is highly dependent on the specific cellular context. FN-1 (Fibronectin 1) is generally considered an oncogene promoting cell adhesion, migration, and invasion in ovarian and other cancers [28, 29]. Similarly, CXCL8 (C-X-C motif chemokine ligand 8) is typically associated with tumor progression, angiogenesis, and metastasis [30].

This apparent contradiction warrants a multifaceted consideration of miRNA regulatory complexities. Firstly, the functional impact of a miRNA can be highly dependent on the specific cellular context, including the tissue of origin and the unique molecular landscape of different cancer subtypes [31, 32]. The distinct characteristics of OVCAR-3 (derived from high-grade serous carcinoma) versus SKOV3 (non-serous carcinoma) cells could therefore dictate differential downstream effects of miR-101-3p, potentially underlying the inconsistent regulation of FN-1 mRNA observed between these two cell lines (downregulated in SKOV3, upregulated in OVCAR-3). Furthermore, microRNAs are known to exert pleiotropic effects by fine-tuning the expression of hundreds of target genes simultaneously, rather than acting as simple on-off switches for individual genes [33, 34]. Consequently, the overall phenotypic outcome—such as the inhibition of migration and invasion in OVCAR-3 cells—is likely the integrated result of miR-101-3p modulating a broad network of targets. In this scenario, the potent downregulation of key pro-metastatic factors by miR-101-3p may collectively override any potential pro-tumorigenic contributions from the incidentally upregulated FN-1 and CXCL8 mRNA. The upregulation of FN-1 and CXCL8 mRNA might also arise from indirect regulatory mechanisms. While miRNAs predominantly mediate gene silencing, they can indirectly cause gene upregulation, for example, by repressing a transcriptional repressor or an mRNA stability inhibitor of FN-1 or CXCL8 [35]. Such complex interactions within miRNA-mRNA regulatory networks are increasingly recognized.

To strengthen this interpretation, it should be noted that miRNAs can also inhibit protein expression predominantly through translational repression rather than mRNA degradation [36, 37]. Thus, the upregulation of FN-1 and CXCL8 mRNA in OVCAR-3 cells does not necessarily imply increased protein activity. The observed anti-invasive phenotype strongly suggests that miR-101-3p suppresses tumor progression through translational repression of FN-1, CXCL8, and/or other more dominant pro-metastatic factors. Moreover, a pathway-centric perspective highlights that miR-101-3p may exert its tumor-suppressive role by converging on key signaling cascades, such as PI3K–AKT and Wnt pathways, which are strongly implicated in ovarian cancer progression. These pathways could represent central nodes that reconcile the seemingly discordant regulation of FN-1 and CXCL8 between different ovarian cancer cell lines.

This study has limitations. We did not assess FN-1 and CXCL8 protein levels, nor did we functionally dissect whether their mRNA upregulation directly contributed to or counteracted the observed phenotype. The precise targets through which miR-101-3p exerts its dominant anti-invasive effects in OVCAR-3 cells, despite the FN-1/CXCL8 mRNA upregulation, remain to be fully elucidated. Future investigations should focus on quantifying protein levels and activities of FN-1 and CXCL8, identifying the direct and critical targets of miR-101-3p in different ovarian cancer cell contexts through techniques like Ago2-RIP-Seq and luciferase reporter assays, and validating these findings in vivo. In conclusion, our study reinforces the tumor-suppressive role of miR-101-3p in ovarian cancer cells by inhibiting their viability, migration, and invasion. However, the paradoxical upregulation of FN-1 and CXCL8 mRNA in OVCAR-3 cells highlights the complexity and cell-type specificity of miRNA-mediated regulation. These findings underscore the necessity for a deeper, context-specific understanding of miR-101-3p’s molecular interactions before its full therapeutic potential in ovarian cancer can be realized.

## Conclusion

In conclusion, our study reinforces the tumor-suppressive role of miR-101-3p in ovarian cancer cells by inhibiting their viability, migration, and invasion. We further show that miR-101-3p modulates distinct downstream targets, including FN-1 and CXCL8, in a cell-type-specific manner, reflecting the complexity of miRNA regulation. Importantly, these findings highlight the potential of miR-101-3p as a therapeutic target in ovarian cancer and underscore the need for future translational research to clarify its context-specific molecular interactions and clinical applicability.

## Supplementary Information

Supplementary Material 1. (A) Venn diagram showing the overlap of upregulated differentially expressed genes (DEGs) between OVCAR-3 and SKOV3 cell lines. (B) Venn diagram showing the overlap of downregulated DEGs between OVCAR-3 and SKOV3 cell lines.

Supplementary Material 2.(A) Gene Ontology (GO) functional enrichment analysis and (B) KEGG pathway enrichment analysis based on overlapping DEGs between the two cell lines.

Supplementary Material 3.Protein–protein interaction (PPI) network analysis of DEGs identified in OVCAR-3 and SKOV3 cells.s

Supplementary Material 4.Kaplan–Meier survival analysis of FN-1 (A) and CXCL8 (B) expression in ovarian cancer patients using the Kaplan–Meier Plotter database.

Supplementary Material 5.List of differentially expressed genes (DEGs) identified by RNA-seq.(A) DEGs between OVCAR-3 cells: control/NC group (A group) vs. mimic group (C group).(B) DEGs between SKOV3 cells: control/NC group (D group) vs. mimic group (F group).Predicted target genes of hsa-miR-101-3p identified using TargetScan, miRDB, and TarBase databases.

## Data Availability

All data collected or assessed in the current study are included in this article. The raw sequencing data generated using high-throughput sequencing have been deposited in the NCBI Sequence Read Archive under the accession number PRJNA1174342 (https://www.ncbi.nlm.nih.gov/sra/PRJNA1174342).

## Abbreviations

NC: Negative control
EMT: Epithelial-mesenchymal transition
DEGs: Differentially expressed genes
