# Supplementary figures and images for: miR-101-3p represses the migratory and invasive abilities of ovarian cancer cells
Source: BMC Cancer. 2025 Dec 11;25:1864. doi: 10.1186/s12885-025-15280-9 (PMC12696907; doi:10.1186/s12885-025-15280-9)

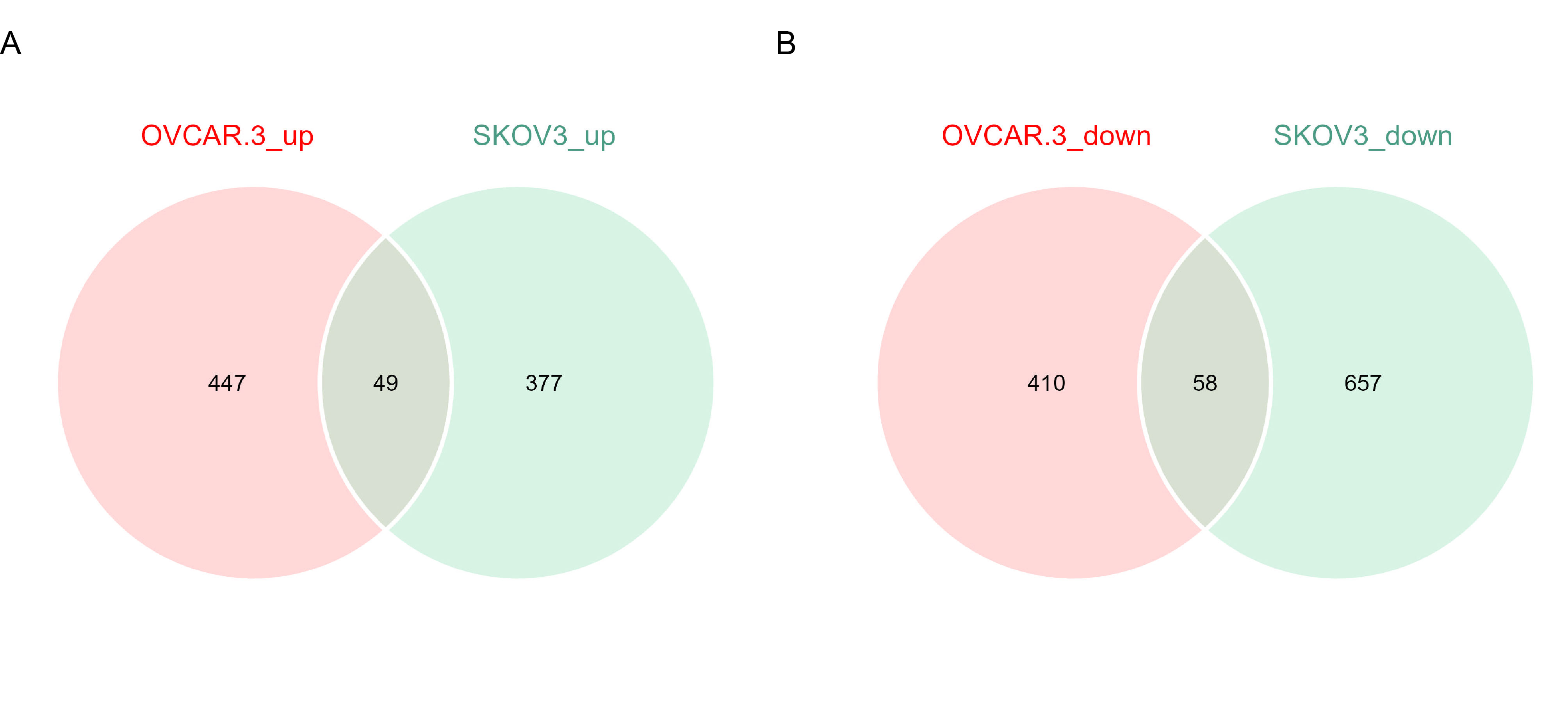

Supplement: Supplementary file 2 — Supplementary Material 2.(A) Gene Ontology (GO) functional enrichment analysis and (B) KEGG pathway enrichment analysis based on overlapping DEGs between the two cell lines. [file 12885_2025_15280_MOESM2_ESM.jpg]

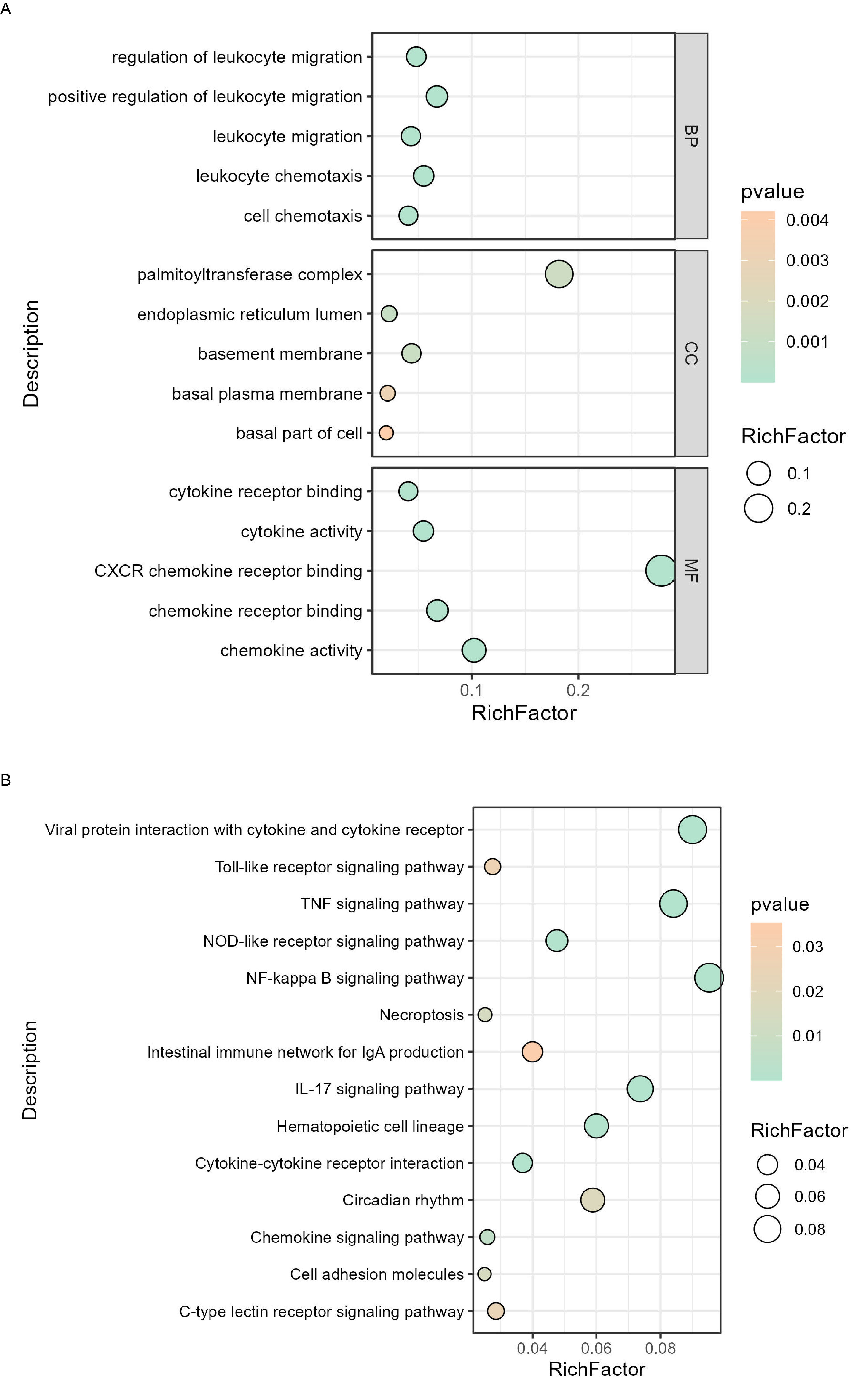

Supplement: Supplementary file 3 — Supplementary Material 3.Protein–protein interaction (PPI) network analysis of DEGs identified in OVCAR-3 and SKOV3 cells.s [file 12885_2025_15280_MOESM3_ESM.jpg]

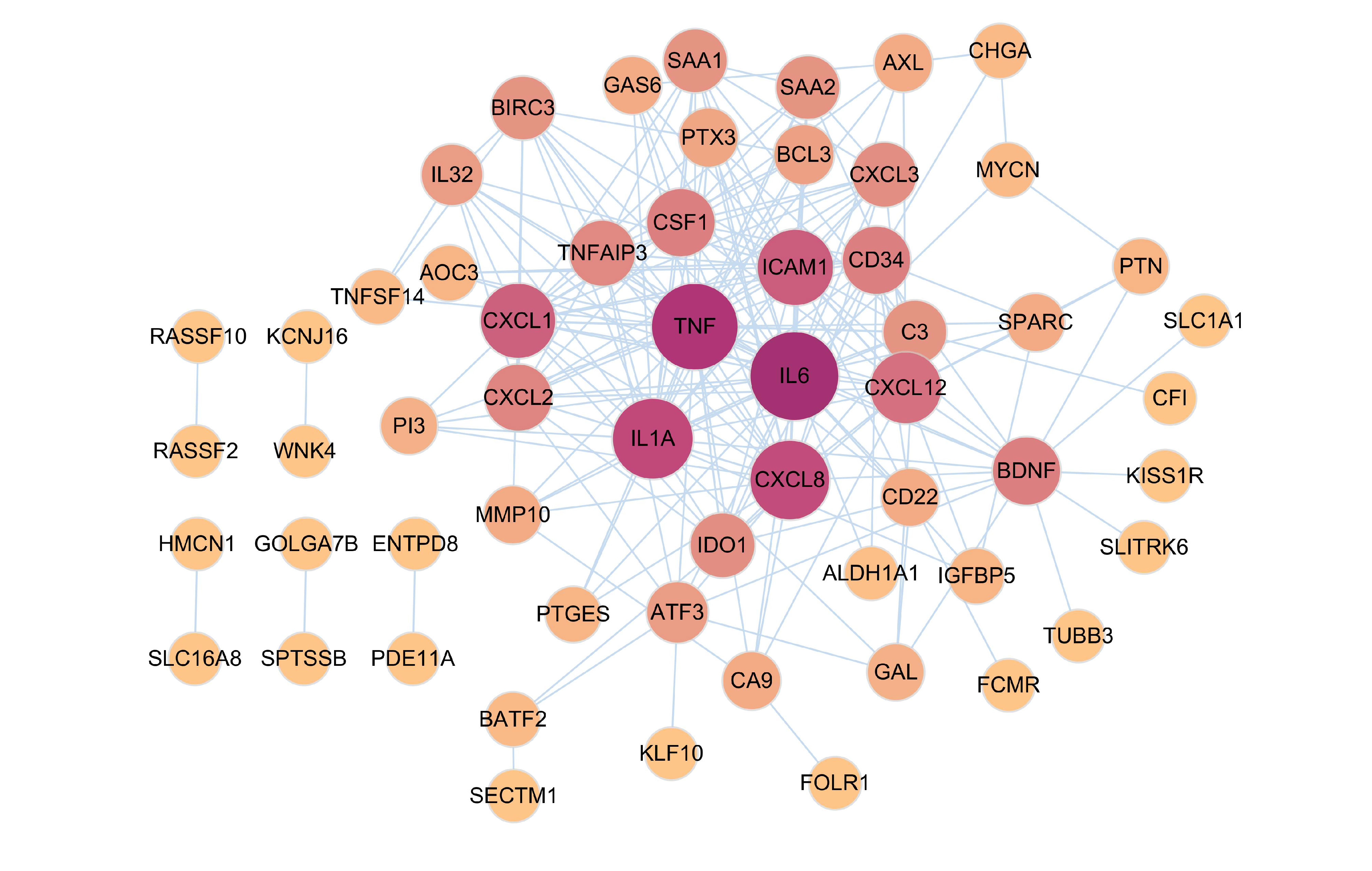

Supplement: Supplementary file 4 — Supplementary Material 4.Kaplan–Meier survival analysis of FN-1 (A) and CXCL8 (B) expression in ovarian cancer patients using the Kaplan–Meier Plotter database. [file 12885_2025_15280_MOESM4_ESM.jpg]

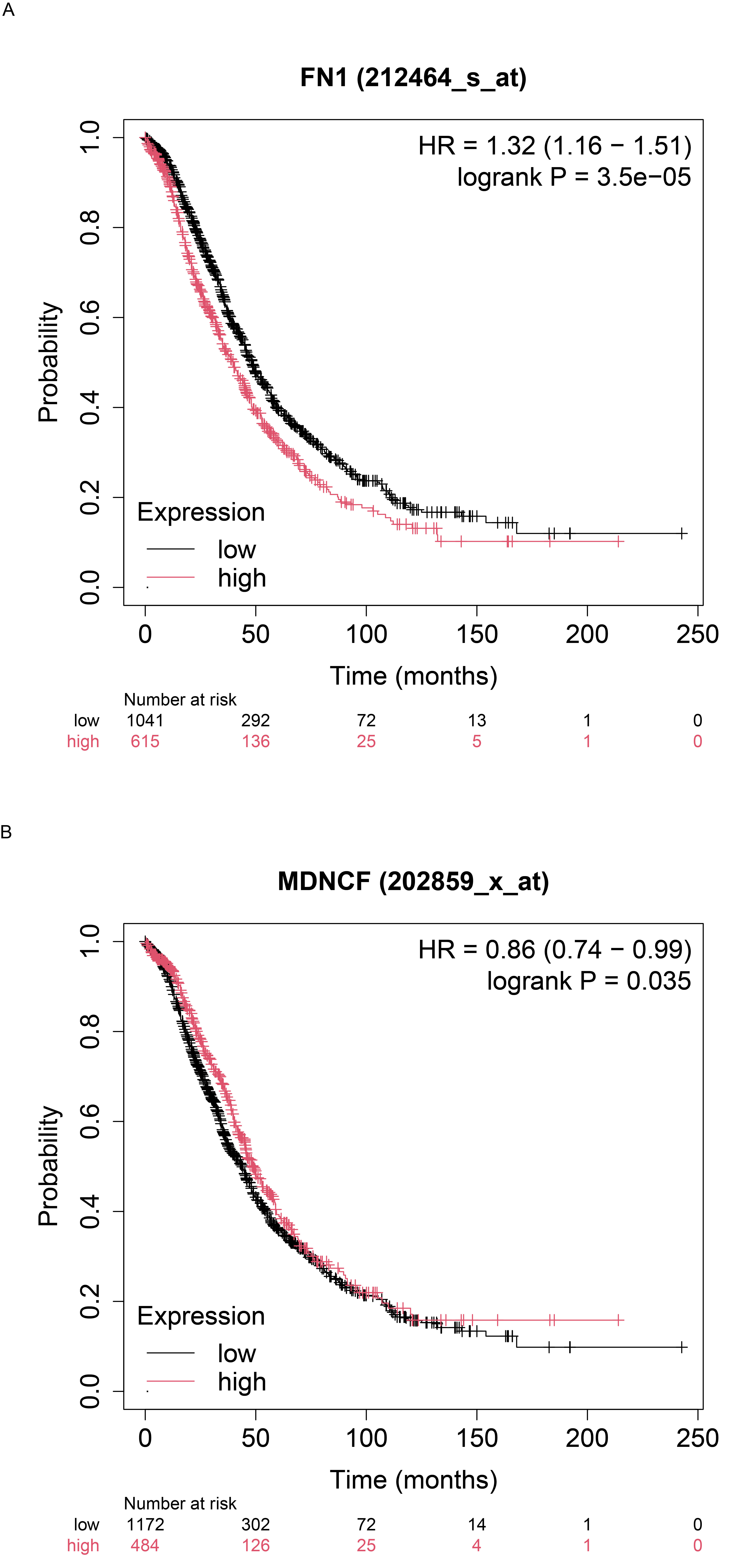

Supplement: Supplementary file 5 — Supplementary Material 5.List of differentially expressed genes (DEGs) identified by RNA-seq.(A) DEGs between OVCAR-3 cells: control/NC group (A group) vs. mimic group (C group).(B) DEGs between SKOV3 cells: control/NC group (D group) vs. mimic group (F group).Predicted target genes of hsa-miR-101-3p identified using TargetScan, miRDB, and TarBase databases. [file 12885_2025_15280_MOESM5_ESM.jpg]
